# Transcriptome Analyses and Antioxidant Activity Profiling Reveal the Role of a Lignin-Derived Biostimulant Seed Treatment in Enhancing Heat Stress Tolerance in Soybean

**DOI:** 10.3390/plants9101308

**Published:** 2020-10-02

**Authors:** Cristina Campobenedetto, Giuseppe Mannino, Chiara Agliassa, Alberto Acquadro, Valeria Contartese, Christian Garabello, Cinzia Margherita Bertea

**Affiliations:** 1 Department of Life Sciences and Systems Biology, University of Torino, 10123 Turin, Italy; cristina.campobenedetto@unito.it (C.C.); giuseppe.mannino@unito.it (G.M.); 2 Green Has Italia S.p.A, 12043 Canale, Italy; v.contartese@greenhasitalia.com (V.C.); c.garabello@greenhasitalia.com (C.G.); 3 Department of Agriculture, Forest and Food Sciences, University of Torino, 10095 Grugliasco (TO), Italy; chiara.agliassa@unito.it (C.A.); alberto.acquadro@unito.it (A.A.)

**Keywords:** seed germination, biostimulant treatment, RNA-Seq, ROS scavenging enzymes and molecules, *Glycine max*

## Abstract

Soybean (*Glycine max* Merr.) is a worldwide important legume crop, whose growth and yield are negatively affected by heat stress at germination time. Here, we tested the role of a biostimulant based on lignin derivatives, plant-derived amino acids, and molybdenum in enhancing soybean heat stress tolerance when applied on seeds. After treatment with the biostimulant at 35 °C, the seed biometric parameters were positively influenced after 24 h, meanwhile, germination percentage was increased after 72 h (+10%). RNA-Seq analyses revealed a modulation of 879 genes (51 upregulated and 828 downregulated) in biostimulant-treated seeds as compared with the control, at 24 h after incubation at 35 °C. Surprisingly, more than 33% of upregulated genes encoded for ribosomal RNA (rRNA) methyltransferases and proteins involved in the ribosome assembly, acting in a specific protein network. Conversely, the downregulated genes were involved in stress response, hormone signaling, and primary metabolism. Finally, from a biochemical point of view, the dramatic H_2_O_2_ reduction 40%) correlated to a strong increase in non-protein thiols (+150%), suggested a lower oxidative stress level in biostimulant-treated seeds, at 24 h after incubation at 35 °C. Our results provide insights on the biostimulant mechanism of action and on its application for seed treatments to improve heat stress tolerance during germination.

## 1. Introduction

Soybean (*Glycine max* L. Merr.) is one of the crops most largely cultivated all around the world, with a strong production located in Brazil, Argentina, and USA [1]. Its market demand has been growing due to increased population and to different applications of this crop. Thanks to its high protein content (40–42%) as compared with other food crops, soybean represents one of the main protein sources for food and feed. Moreover, it is also largely employed for oil production, owing to the fact that it has one of the highest oil contents (18–20%) among legume crops [1]. For all these reasons, it has become essential to optimize its production, and avoid to use new lands, which would lead to deforestation phenomena or to the subtraction of arable land devoted to the cultivation of other crops [2]. One of the most important limiting factors affecting soybean growth and yield is represented by abiotic stresses, especially heat stress, which is a negative feature common in the regions in which this crop is currently cultivated [3]. Soybean, as compared with other crops, is quite tolerant to high temperatures, with an optimum around 30 °C during vegetative growth. Contrarily, seed germination, along with seed development, represent critical phases in which the optimal temperature range is about 22–24 °C [4,5,6]. A high temperature during this phase inhibits seed germination, and also slows down growth and reduces yield and quality [4,5,6]. So far, different approaches have been employed aimed to enhance plant stress tolerance, however, some treatments may be particularly time-consuming (e.g., conventional breeding) or not universally accepted by all countries (e.g., plant genetic modification). Consequently, it has become fundamental to help plants to resist abiotic stresses by using new and faster alternative solutions, which should be cost effective and less dependent on ethical issues [7]. In order to enhance germination rate and growth homogeneity, seed treatments can be used. These techniques consist of a partial hydration of the seeds followed by a dehydration step, which aim at activating various processes without completing germination [8]. In this way, once the seeds are sown under normal or stress conditions, they exhibit a faster and more homogeneous germination, together with a higher tolerance to stresses. Similar to priming treatments, seed treatments make the seed more responsive to external stimuli and able to counteract stress conditions before seedling emergence [7,9]. So far, in the literature there are few references about biostimulants used for these purposes [10,11,12,13]. Biostimulants, which are a new generation of products that have been available on the market for a few years, could play a role as seed treatment agents [14,15]. In particular, they have received considerable attention due to their wide range of biological activities, and in particular for their effect against abiotic stress [16,17,18]. Abiotic stresses, including heat stress, strongly affect the yield and quality of production, influencing seed germination, as well as plant growth and development [15]. In particular, final crop yield can be strongly compromised if the stress occurs during the most sensitive phenological phases [15]. In this context, seed treatments using biostimulants have been proposed as agronomic tools to counteract the negative effects of abiotic stresses. Although the action mechanisms of these products are not well known, scientific evidences suggest that the beneficial properties are linked to their content in bioactive molecules, which are able to improve a plant’s capability to face adverse environmental conditions, affecting the primary or secondary metabolism [9,19,20].

In this study, we evaluated the efficiency of a seed treatment with a biostimulant based on lignin derivatives (lignosulphonates) that also contain plant-derived amino acids and molybdenum [9], for promoting soybean tolerance to heat stress. This product was especially studied for the Brazilian market, since, in this country, adverse conditions at sowing time are often present. Degenerative changes in soybean seeds are mainly caused by alternating periods of dry and moist conditions, and are they are also associated with high temperatures before germination [21]. In this context, more recently, the Brazilian area has experienced an increase in the annual temperature of about 0.5 °C, together with an intensification of rainfall of about 4% [22]. Temperature and rainfall have a crucial effect on soybean growth processes. Droughts during flowering, grain and filling, and maturation cause a reduction in yield due to higher flower abortion, following by a reduction in the flowering period, amount of grain, grain filling period, and grain quality [23]. High temperature causes disturbances in flowering and reduces the retention capacity of pods [23].

In this work, a biostimulant was tested on soybean in order to evaluate its effects on seed germination and plant development under heat stress conditions. The analyzed biometric and morphological parameters included seed early germination and development under controlled conditions. In order to determine the effects and the metabolic targets of this product, morphological, RNA-Seq, and biochemical (ROS-scavenging system) analyses were carried out on biostimulant-treated seeds, incubated for 24 h at 35 °C, in the dark, and compared to untreated seeds grown in the same experimental conditions. All the results taken together provide insights on the mechanism of action of the biostimulant and on its application as a seed biostimulant able to increase tolerance to heat stress.

## 2. Results

### 2.1. Biostimulant Application Positively Influenced Seed Morphological Parameters and Germination Percentage under Controlled Conditions

In order to evaluate whether the biostimulant treatment had a visible influence on soybean seed morphology at the early germination phase under heat stress conditions, experiments were carried out under controlled conditions. The biometric parameters were monitored on both control and biostimulant-treated seeds, at the start of treatment (T_0_) and at 24 h, after incubation at 35 °C, in the dark (T_1_). Except for the weight, all the evaluated parameters showed a significantly higher percentage increase in seeds treated with the biostimulant as compared with untreated seeds (Table 1). The biostimulant treatment prompted a significant increase in seed area (+50.34%), perimeter (+18.28%), length (+20.39%) and width (+14.74%) as compared with the control. By contrast, the weight increase was only 0.63% (not statistically significant) higher in biostimulant-treated seeds as compared to control seeds.

The effect of the biostimulant application on soybean seed germination is shown in Table 2. The germination percentage of control and biostimulant-treated seeds was recorded at 24, 48, and 72 h after incubation at 35 °C in the dark. The biostimulant treatment prompted a significant increase in the germination percentage as compared with the control, both at 48 and 72 h. In addition, at 24 h a visible germination was not detected.

### 2.2. Biostimulant Application Modulated the Expression of 879 Genes under Controlled Conditions

**Figure 1:**
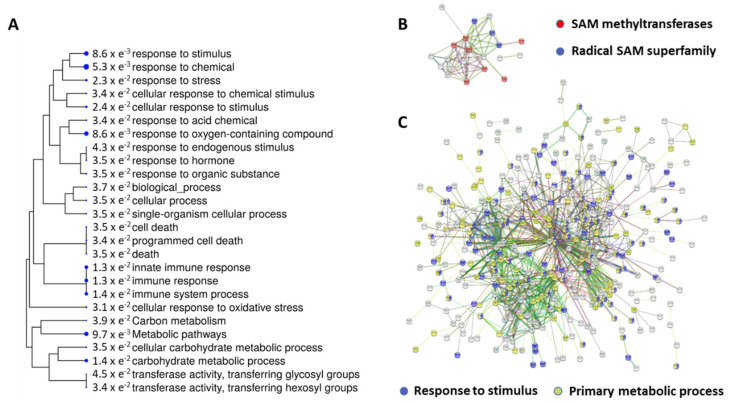
(**A**) Graphical representation of summarized tree of the enriched gene ontology (GO) terms; (**B**) Functional interactions among the upregulated genes; (**C**) Functional interactions among the downregulated genes. ShinyGO^®^ generated the hierarchical clustering tree, using the significant GO terms listed in Table 3. GO terms with many shared genes are clustered together. Bigger dots indicate more significant *p*-values. The graphical representations of the functional interactions were generated by STRING software.

A total of 879 genes were differentially expressed in biostimulant-treated seeds with respect to untreated seeds at 24 h, after incubation at 35 °C in the dark. Among all the genes, 51 were upregulated, meanwhile 828 were downregulated by the treatment. The list of modulated genes is reported in Supplementary Table S3, whereas the Volcano plot generated by the RNA-Seq analysis is shown in Supplementary Figure S1. A hierarchical clustering tree, summarizing the correlation among significant GO terms was generated (Figure 1A).

The upregulated genes (Table 3) included in particular methyltransferases, genes involved in different processes such as DNA repair, molecule biosynthesis, protection from enzymatic degradation, and response to abiotic stress [24,25]. Among them, the most represented families were the adenosyl-L-methionine-dependent methyltransferase superfamily [IPR029063, 9 genes, FDR (False Discovery Rate) = 6.65 × e^−8^), the Class A methyltransferases (IPR040072, 3 genes, FDR = 8.76 × e^−5^), and ribosomal RNA (rRNA) processing proteins (KW-0698, 4 genes, FDR = 3.70 × e^−5^).

However, most of the genes were downregulated (Table 3), and many of them were related to response to stress (GO:0006950, 80 genes, FDR = 5.49 × e^−06^). In particular, treated seeds showed a reduced expression of genes coding for heat shock transcription factors, such as *LOC100527682*, *LOC100786140,* and *LOC100789792*. These genes are also part of two other GOs as follows: response to chemical (GO:0070887, 90 genes, FDR = 8.83 × e^−11^) and response to stimulus (GO:0050896, 135 genes, FDR = 2.24 × e^−10^). Moreover, these GOs grouped genes coding for enzymes involved in the redox regulation, such as glutathione-S-transferase (*LOC100808374*), glutaredoxins (*LOC100792704*), and thioredoxins (*LOC100810192*, *LOC100800129*). Among the downregulated genes, we also found some responding to chemical, such as peroxidases 3 (*LOC100547872*) and peroxidase 5 (*LOC100811641*), which are involved in removal of H_2_O_2_, oxidation of toxic reductants, biosynthesis and degradation of lignin, suberification, auxin catabolism, and response to environmental stresses. In the same category, the biostimulant treatment downregulated the expression of genes coding for a protein kinase superfamily protein (*LOC100794703*) which acts as a positive regulator of abiotic stress response and zinc finger transcription factors (*LOC100806997*).

The biostimulant treatment appeared to influence the expression of genes coding for repressors of hormone signaling, such as AUX/IAA (Auxin/Indole-3-Acetic Acid) proteins (*LOC100802759*, *LOC100791342*, and *LOC100799875*) and DELLA (aspartic acid–glutamic acid–leucine–leucine–alanine) proteins (*LOC100805968* and *LOC100791952*), together with protein kinases (*LOC100794703*) and receptor kinases (*LOC102668647*) acting into hormone-induced pathways. Along with a general downregulation of stress-related responses and the influence on hormone signaling, the biosynthesis of secondary metabolites (Kegg pathway 01110, 10 genes, FDR = 0.000697) was decreased in the presence of the biostimulant. Among the others, the biostimulant treatment downregulated genes coding for cytochrome P450s and enzymes such as beta glucosidases (*LOC100820528*, *LOC100777773*) and O-methyltransferases (*LOC100787536*) which act on phenolic compounds. The GO analysis also highlighted a global downregulation of primary metabolic processes (GO:0044238, 160 genes, FDR = 0.000933), including carbohydrate metabolic process (GO:0005975, 35 genes, FDR = 0.00117). In particular, it regulated the expression of genes which encode for enzymes involved in the promotion of cell wall organization or biogenesis (GO: 0071554, 21 genes, FDR = 0.00993) such as pectinesterases (i.e., *LOC100792319*, *LOC100794948*), glucosyl transferases (*LOC100812586*), and xyloglucan:xyloglucosyl transferases (*LOC100778482*). Moreover, the biostimulant acted on genes involved in sucrose metabolism such as sucrose synthase 4 (*LOC100819730*), and trehalose accumulation such as haloacid dehalogenase-like hydrolase domain-containing protein and trehalose-6-phosphate phosphatase J (*LOC100803119*). The glycolysis and fermentation pathways were also affected by the biostimulant treatment since both phosphofructokinase 3 (*LOC100818755*) and alcohol dehydrogenase 1 (*LOC100800668*) transcript levels were reduced. The enriched primary metabolic process GO term (GO:0044238) also grouped genes involved in protein and amino acid metabolism. In particular, our analysis showed the downregulation of glutamate decarboxylases (*LOC100812201* and *LOC100781791*) and aspartate aminotransferases (*LOC100780254*), able to catalyze the biosynthesis of γ-aminobutyric acid (GABA) and aromatic amino acids, respectively [26,27]. Last, but not least, lipid metabolism was also negatively regulated by the biostimulant. For instance, the primary metabolic process GO term (GO:0044238) also contained lipid phosphate phosphatase 2 (*LOC100782531*), long-chain acyl-CoA synthetase 2 (*LOC100806645*), and lipoxygenase 4 (*LOC100811820*).

### 2.3. String Suite Analysis Revealed Two Functional Interaction Networks

In order to identify the cellular functions affected by the biostimulant treatment, and to support data analysis, all the functional interactions between the expressed proteins were investigated in both up- and downregulated datasets and STRING suite (Figure 1). Regarding the interactions of the upregulated genes, a number of 34 nodes and 63 edges was observed (average node degree = 3.71) (Figure 1B). Considering the value of 12 as the expected number of edges for 34 nodes, this network had significantly more interactions than expected [PPI (Protein-Protein Interaction) enrichment *p*-value = 1.00 × e^−16^]. A clear, but small, network was observed, related to SAM-dependent methyltransferase and SAM superfamily. Considering the interactions of the downregulated genes (Figure 1C), 483 nodes and 1078 edges were observed (average node degree = 4.46). In addition, this network had significant enrichments (PPI enrichment *p*-value = 1.00 × e^−16^), 594 being the expected number of edges for 483 nodes. In this dataset, the visible highly interconnected subgroups were those linked to stimuli response and those linked to primary metabolism.

### 2.4. Biostimulant Application Reduced Oxidative Menaces

**Figure 2:**
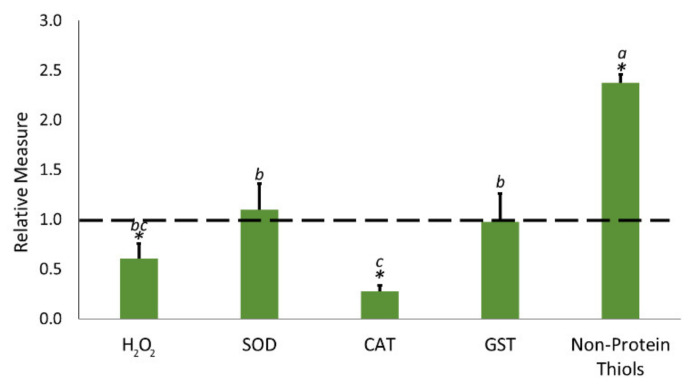
Effects of the application of the biostimulant on hydrogen peroxide (H_2_O_2_) level, superoxide dismutase (SOD), catalase (CAT), and glutathione S-transferase (GST) enzymatic activity and on non-protein thiol content. Data were calculated as relative measure, by comparing each measure recorded on the biostimulant-treated sample to the related untreated sample (dotted line). The individual raw measures for each parameter are reported in Supplementary Table S4. Each bar represents the mean ± standard deviation (SD). Lowercase letters indicate significant (*p* < 0.05) differences among the different relative measures, as calculated by one-way ANOVA followed by Tukey’s test. The asterisk (*) indicates statistical differences between untreated (dotted line) and treated samples, as calculated by *t*-test (*p* < 0.05). Additional statistical information is reported in Supplementary Table S5.

In order to gain more insight into the seed response to heat stress and H_2_O_2_ production during the early phases of seed germination, the level of H_2_O_2_, the activities of ROS scavenging enzymes (superoxide dismutase (SOD), catalase (CAT) and glutathione S-transferase (GST)), and the content of non-protein thiols were analyzed after the application of the biostimulant. The obtained data were compared with those measured in non-treated samples, and results were expressed as relative value (Figure 2). Supplementary Table S4 provides the report of the absolute values of this quantification. In general, the biochemical analysis conducted on biostimulant-treated soybean seeds at 35 °C suggested a better response to stress as compared with the control (untreated seeds). However, it seemed that the capability to contrast oxidative menaces, apparently, was not linked to changes in enzymatic activity. Indeed, after the application of the biostimulant, the enzymatic activity of SOD and GST did not show significant differences with respect to untreated seeds, and CAT activity was strongly downregulated (FC 0.28). In addition, the content of H_2_O_2_ was drastically reduced (−40%) and the content of non-protein thiols strongly increased (+150%).

## 3. Discussion

Seed treatment technologies consist of seed treatment with synthetic or natural compounds, which are aimed at enhancing the uniformity and vigor of seedlings and improving the plant tolerance to abiotic stresses [28]. The use of biostimulants to counteract the effect of abiotic stress is well-recognized and their function in promoting plant defenses against adverse environmental conditions has been studying lately [9,12]. Seed pretreatments have been revealed as a good method to contrast the ever-increasing environmental stress presence, improving the yield of productions, starting from seed germination [29]. This is a faster method as compared with conventional breeding or plant genetic modification and could be useful for seed treatment in countries, such as Brazil, where high temperature at sowing represents a limiting factor [30]. Among the crops whose cultivation occurs under this adverse environmental conditions, soybean (*Glycine max*) is affected in terms of poor germination, increased incidence of pathogen infection, and decreased economic value [31].

The potential effects derived from the application of the biostimulant objective of this study, was preliminary investigated, in 2017, at the Detec Experimental Station located in Brazil (Taquarituba, 23°31’33.7"S 49°15’16.6"W) (Supplementary Table S1). During these field trials, the biostimulant applied as seed treatment on soybean, was able to positively affect plant vegetative and reproductive growth under natural heat stress conditions. The biostimulant seed treatment efficiency for improving germination and mitigating oxidative damages under heat stress conditions has been observed in our previous work on cucumber seeds [9]. In the present work, in order to evaluate the potential effects of the biostimulant in promoting heat stress tolerance also in soybean, we conducted germination trials under controlled conditions, and we investigated the potential mechanism of action using combined biochemical and transcriptomic (RNA-Seq) approaches.

### 3.1. The Biostimulant Promoted Soybean Germination and Growth after Heat Stress Exposure

Our preliminary field trials showed that at seven days after sowing, root length and height of plants grown from biostimulant-treated seeds were higher with respect to the control plants (Supplementary Table S1). Moreover, the final yield observed in plants primed by the biostimulant was generally higher at harvesting time, suggesting that the pretreatment effect was prolonged at later plant growth stages. Indeed, although the biostimulant seemed not to significantly change soybean germination rate at the early stage, the final germination percentage measured under controlled conditions, appeared to be higher in biostimulant-treated seeds at 72 h after incubation at 35 °C. Likewise, the biostimulant seed treatment increased cucumber germination percentage under heat stress conditions only at 48 h after incubation at 35 °C [9]. Therefore, our results give an indication of a possible late effect of the biostimulant in promoting plant vegetative, and then reproductive growth, under heat stress conditions [32].

It has been demonstrated that in salt stress, the activation of different biochemical mechanisms related to stress response, promotes a ”priming memory” in seeds which can be recruited upon a later stress exposure and provokes higher stress tolerance of germinating primed seeds [33]. Wei and co-workers observed that seven-day-old soybean seedlings grown from melatonin-treated seeds and subjected to drought stress showed an enhanced tolerance as compared with untreated seeds [34]. In accordance with our results, treatment of maize seeds with solutions of increasing concentrations of lignin carbon showed no influence on seed germination rate, but a positive biological activity on the emergence of maize plantlets [35]. Moreover, Amirkhani and co-workers showed that broccoli seeds coated with plant protein lysates enhanced seedling shoot and root growth as compared with uncoated seeds, whereas their germination was negatively affected by the treatment [36]. This negative effect could be related to a barrier for water uptake and gas exchange due to the coating during the whole germination process. Interestingly, the biostimulant also contains lignin derivatives and protein hydrolysates of plant origin. In addition, our data do not show any differences at the weight level for primed seeds at 24 h after incubation at 35 °C, thus suggesting the absence of changes in water uptake. In parallel, our RNA-Seq data analysis showed a downregulation of aquaporin gene TIP2-1 (*LOC100803901*). Aquaporins are proteinaceous channels known to regulate transmembrane water transport, and therefore play a key role in the imbibition process during seed germination [37]. Previous studies conducted on spinach (*Spinacia oleracea* L.) unprimed and osmoprimed seeds subjected to chilling and drought stress showed a downregulation of the genes coding for aquaporins [including TIP (Tonoplast Intrinsic Protein) subfamily] as compared with seeds germinated in optimal conditions [38]. There is evidence that different seed pretreatments cause a decrease in water absorption owing to the lower water potential of the solution. In this way, the treated seeds have more time to complete DNA repair processes and also reduce cellular damage, which often occurs during rapid seed rehydration in the germination process [30]. This hypothesis is also supported by RNA-Seq analysis, and in particular from the upregulation of Elongator protein 3/MiaB/NifB, SAM, and FTSJ-like methyltransferase genes (Table 3).

In addition to aquaporin downregulation, we could not exclude that the differences observed for some biometric parameters such as area, perimeter, length, and width of the seeds treated with the biostimulant could be related to a different level of gas exchange. Indeed, in the literature, seed pretreatments have been reported to induce the formation of a thick mucilage layer during imbibition, which could affect water and gas exchanges, by promoting seed swelling without increasing the seed weight [14,39]. However, this hypothesis should be confirmed in future experiments.

### 3.2. Rna-Seq Analysis Displayed That Heat Stress Mitigation after Biostimulant-Treatment May Be Linked to Different Molecular Pathways

Although the biostimulant treatment did not affect the early germination percentage, RNA-Seq analysis seemed to corroborate the already active effect of the biostimulant for mitigating stress in seeds germinated under high temperature conditions. First, we observed a strong downregulation following biostimulant treatment, since only 51 genes out of 879 modulated, were upregulated (Table 2 and Table 3). However, a stronger downregulation, as compared with upregulation, was observed with other biostimulants. For example, the treatment with lipophilic components of the brown seaweed *Ascophyllum nodosum* improved freezing tolerance in *Arabidopsis thaliana* by inducing the upregulation of 463 genes and the downregulation of 650 genes [40]. Moreover, Contartese et al. (2016) similarly observed a higher number of downregulated genes (2415) as compared with the upregulated genes (1731) in tomato plants treated with Expando^®^, a biostimulant developed to increase fruit size [41].

In this study, in the presence of heat stress, soybean seeds treated with the biostimulant showed upregulation of three proteins involved in the biogenesis of ribosomes, as well as a specific group of 12 methyltransferases acting on rRNA (Table 4, Figure 1B). DNA methylation is well known to affect plant developmental processes and to be activated in response to abiotic stress [42], strongly connected to the perception of external environmental stimuli, to changes in stress-responsive gene expression, and to pretreatment effect activation [43,44]. In contrast, RNA methylation in plants is less investigated, but accumulating reports have demonstrated that RNA methyltransferases are essential for plant growth as well as for abiotic stress responses [45]. In animals, ribosomal RNAs (rRNA), which are encoded by multiple copies of rDNA at the genomic level, display tissue-specific expression patterns, raising the chance to create combinations of rRNAs and ribosomal proteins forming ribosomes (“ribosome heterogeneity”) likely able to translate subsets of transcripts [46]. Ribosomal RNA modifications (e.g., methylation) during production/maturation are at the base of the ribosome heterogeneity [46] which can regulate ribosome assembly on a group of specific transcripts [47,48]. Recently, a specific ribosome methylation event facilitating the selective translation of a cyp-29A3 gene has been reported, which by increasing, the production of eicosanoids, modulated stress resistance in *C. elegans* [49]. In our system, the heat stressed seeds, pretreated with the biostimulant, specifically induced rRNA methyltransferases which exhibited a high level of connectivity in the same emerging gene network (Figure 1B). According to [49], this methylation process could, by analogy, facilitate selective translation of genes guiding the observed resistance to heat stress induced by the biostimulant treatment. This hypothesized translation-dependent layer of regulation deserves to be deeply investigated in future experiments.

Moreover, few genes involved in stress response (Supplementary Table S3) are also upregulated by the application of the biostimulant. In particular, our analysis revealed the upregulation of NF-X Like 1 (*LOC102669482*) and pentatricopeptide repeat containing protein (*LOC100788313*). On the one hand, the human NF-X1 protein and homologous proteins in eukaryotes represent a class of transcription factors whose common feature is the cysteine-rich region, which possess a variable number of repeated motifs, defined as NF-X1 type zinc finger motifs. It has been demonstrated in Arabidopsis that NF-X Like 1 protein is involved in a regulatory mechanism able to improve the physiological status of plants and to support growth and survival under salt stress [50]. On the other hand, pentatricopeptide repeat (PPRs) constitutes one of the largest gene families in Arabidopsis. Several genes belonging to this family are involved in tolerance to different biotic and abiotic stresses [51]. The mitochondrial PPR protein PGN (pentatricopeptide repeat protein for germination on NaCl) was identified to positively regulate biotic and abiotic stress response. Arabidopsis plants with mutation in PGN show low resistance against necrotrophic fungi as well as towards GABA, glucose, and high salinity [52].

The potential stress mitigation induced by the biostimulant treatment could also be confirmed by the downregulation of stress response-related genes whose interconnection is high in a protein-protein network (Figure 2). Among others, genes coding for heat shock factors (HSFs), in particular HSFB2A, B3, and B4 (*LOC100789792*, *LOC100786140* and *LOC100527682*) were found to be downregulated. Currently, more than 30 genes coding for HSF divided into three classes, A, B and C, have been described in soybean [53]. Functional analyses show that some members of class B HSFs are active transcriptional repressors. The repressive activity of HSFB1 and HSFB2b was reported also in Arabidopsis and a domain, designated the B3 repression domain (BRD), was found in HSFB1 and HSFB2b at the C terminus [54]. The two HSFs, HSFB1 and HSFB2b, are able to repress the general heat shock response under non-heat-stress conditions and act via direct repression of the expression of the heat stress inducible HSFA2 [54]. The overexpression of *OsHsfB2b*, a member of B HSF in rice, significantly decreased drought and salt tolerance in transgenic plants, while the *OsHsfB2b-RNAi* plants enhanced tolerance under these stresses indicating that this protein could negatively regulate the abiotic stress tolerance. The observed downregulation of HSFs belonging to class B could be important for promoting enhancement of heat stress tolerance in the biostimulant-treated seeds germinated at 35 °C.

The RNA-Seq analysis also reveals the downregulation of different genes belonging to redox response, such as different isoforms of peroxidases and RbohB, a gene coding for a calcium-dependent NADPH oxidase that generates superoxide. These proteins are involved in responses to different stresses as revealed by reverse genetics approach and ”overexpression” of *Rboh* genes [55]. For example, the pathogens *Pseudomonas syringae* and *Peronospora parasitica* induce an oxidative burst in Arabidopsis by enhancing *AtrbohD* and *AtrbohF* expression [56]. Interestingly, *Rboh* was also downregulated at 24 h in cucumber seeds pretreated with the biostimulant and incubated at 35 °C [9].

### 3.3. Biostimulant Treatment Mitigated the Accumulation of Reactive Oxygen Species (Ros) via a Non-Enzymatic Way

In seed physiology, reactive oxygen species (ROS) are usually considered to be toxic molecules, whose accumulation leads to cell injury and consequent problems in seed germination and development. However, there is increasing evidence that ROS, at low concentrations, can function as signaling molecules involved in a wide range of responses to various stimuli [57]. The dual function of ROS in plants mainly relies on the cellular antioxidant machinery, which involves detoxifying enzymes and antioxidant compounds. Such mechanisms can scavenge potentially toxic ROS, generally produced under stressful conditions, or rather tightly control ROS concentrations in order to regulate various signaling pathways. Among ROS, hydrogen peroxide plays a key role during germination, however too high levels of H_2_O_2_ can be toxic for seeds [58]. In our study, the level of H_2_O_2_ were significantly lower in the biostimulant-treated seeds, suggesting a possible role of the biostimulant for preventing its accumulation. Biostimulant-treated cucumber seeds also showed a reduction in endogenous H_2_O_2_ levels [9]. The lower amount of H_2_O_2_ observed in biostimulant-treated soybean seeds is correlated to CAT activity. This enzyme, along with peroxidase (PRX), is directly involved in the disruption of H_2_O_2_, and their activation indicates the effort of plants to reduce oxidative damage. A lower activity of CAT and an undetectable activity of PRX can be an indication of the biostimulant’s capability for mitigating stress effects on the seed germination process. Our enzymatic data are confirmed also by gene expression results. Indeed, genes coding for peroxidases, such as PRX-3 and PRX-5, resulted downregulated along with *RbohB* (Table 3). Non-protein thiol compounds represent another important parameter in stress response. They are the most important source of sulfur in soybean seed, a fundamental element involved in metabolic pathways, nutritional quality, and plant productivity. Thiol metabolism plays a key role in plant growth, development, and defense against a range of environmental stresses [59]. It has been demonstrated that thiols, such as glutathione (GSH) together with its regulation in redox signaling and defense processes, are important components for the heat stress tolerance [60]. The glutathione pool has been shown to be associated with the response to heat stress of maize [61], *Coleus blumei* and *Fagus sylvatica* L. [62], *Triticum aestivum* [63], and *Vigna radiata* [64]. Soybean seeds have nearly undetectable levels of glutathione, but this compound is substituted by the tripeptide homoglutathione which functions in a similar way to GSH by showing the same redox reaction [59,65]. Glutathione-S-transferases (GST) are directly linked to thiols, since they play important roles in enzymatic thiol-dependent ROS scavenging mechanisms [66] by catalyzing the conversion of H_2_O_2_ using glutathione or homoglutathione as substrates. In our study, the levels of non-protein thiols were higher in the biostimulant-treated seeds as compared with the untreated seeds, while the activity of GST did not change after biostimulant application. Differently, the biostimulant promoted an upregulation of GST activity in *Cucumis sativus,* at 24 h after incubation at 35 °C, while non-protein thiol level was higher at 48 h [9]. In general, the biostimulant seed treatment led to a reduction in ROS accumulation, due to a direct or indirect scavenging effect induced by the product [67,68]. On the basis of our results, we hypothesize that the biostimulant pretreatment effects on antioxidant capacity appear to be more correlated with non-protein thiol levels than with increased transcription or activity of enzymatic ROS scavengers in soybean seeds.

### 3.4. Soybean Seed Primary Metabolism and Hormone Signaling Are Affected by the Biostimulant Treatment

Surprisingly, the biostimulant treatment also affected the primary metabolism of soybean seeds by inducing a dramatic global downregulation of primary metabolism process (GO:0044238, 160 genes, (Figure 1), whose activation was directly related to the promotion of germination [69]. Concerning carbohydrate metabolism, sucrose synthase is one of the most important enzymes directly involved in the germination process, since it catalyzes the reversible cleavage of sucrose to glucose and fructose. Its downregulation induced by the biostimulant seems to confirm the reduction in the isolation of sugar-nucleotide precursors for structural and storage polysaccharide biosynthesis [70], which indeed appears to be negatively affected by the biostimulant treatment. Moreover, some of the carbohydrate metabolism regulated genes control trehalose accumulation, which has a role in improving the abiotic stress tolerance [71]. Interestingly, the glycolysis pathway is also affected by the biostimulant treatment, i.e., a decrease in the transcript level of phosphofructokinase 3, encoding for the enzyme which catalyzes the phosphorylation of D-fructose 6-phosphate to fructose 1,6-bisphosphate by ATP, the first committing step of glycolysis. Moreover, alcohol dehydrogenase 1 downregulation suggests a reduced alcohol fermentation in seeds treated with the biostimulant. Together with the carbohydrate metabolism, protein and amino acid metabolism is also affected by the biostimulant treatment. Seed storage proteins are critical to provide amino acids for protein synthesis and energy production, particularly glutamate and aspartate, which are the most abundant amino acids in soybean seeds. In particular, our analysis showed the downregulation of glutamate decarboxylases and aspartate aminotransferases, both encoding for enzymes whose activity is promoted by imbibition and whose role is essential along seed germination [69]. While glutamate decarboxylase (*LOC10081220*) promotes the accumulation of GABA [27], aspartate aminotransferase (*LOC100780254*) directly promotes the biosynthesis of aromatic amino acids [72]. Since imbibition is a critical step in seed germination, the downregulation of the primary metabolism genes observed with the biostimulant-treated soybean seeds might be attributable to the lower water absorption linked to the seed treatment process. However, this slowing down in germination has also been demonstrated to be important for DNA repair and cellular damage prevention [73].

The hormone mediated signaling pathway (GO:0009755) has a key role in modulating seed germination, development, and response to stress [74]. In particular, during soybean germination, auxins and ABA (Abscisic Acid) appear to repress germination [75], while gibberellin and ethylene promote germination [69]. Our transcriptomic analysis highlighted a complex influence of the biostimulant treatment on seed hormone cascade. Concerning auxin signaling, the biostimulant treatment appeared to significantly reduce the expression of different AUX/IAA proteins (IAA29, IAA19, and ATAUX2), which are known to act as repressor of early auxin response genes under low auxin concentration [74]. On the contrary, the biostimulant seemed to positively affect the gibberellin signaling pathway by negatively modulating the expression of genes coding for DELLA proteins (*LOC100805968*, GAI and *LOC100791952*, RGL3), which act as repressor of gibberellin signaling. Last but not least, ABA signaling was also regulated by the biostimulant treatment, in terms of decreasing the expression of genes encoding for protein kinases (*LOC100794703*) and receptor kinases (RK1) acting along its signaling pathway.

## 4. Materials and Methods

### 4.1. Plant Material and Biostimulant

For this study, soybean seeds, variety PR91M10, were obtained by Pioneer Hi-Bred Italia Srl (Gadesco Pieve Delmona (CR), Italy), and used for the experimentation in controlled conditions. The treatments were performed using the biostimulant KIEM^®^ (Green Has Italia S.p.A, Canale, CN, Italy). The label of the product claims to contain 2% (*w*/*w*) of organic nitrogen, 2% (*w*/*w*) of molybdenum, and 21% (*w*/*w*) of organic carbon. The pH (1% acq. sol. *w*/*w*) and electrical conductivity (in acq. sol. 1g L^−1^) were also reported and were 4.00 ± 0.5 and 200 µS cm^−1^, respectively. Moreover, the chemical profile of the biostimulant revealed the presence of several amino acids (leucine, isoleucine, threonine, methionine, phenylalanine, alanine, serine, and glutamic acid), organic acids (lactic acid, sulfuric acid, phosphoric acid, and citric acid), sugars (fructose and galactose), myoinositol, oxoproline, and glycerol in detectable amounts [9].

### 4.2. Experiments in Controlled Conditions

Soybean seeds were treated by following the protocol provided by Embrapa in 2005 [76], described in details in Campobenedetto et al. [9], and currently used in Brazil for the seed treatment with different products, including phytochemicals and biostimulants [77]. Briefly, 20 mL of the biostimulant solution was diluted in distilled water in order to reach the final volume of 80 mL. Then, the diluted biostimulant solution was added drop by drop to 250 g of dried seeds kept in continuous shaking, until the complete and visible distribution of the product on the seed surface was obtained. After the treatment, the seeds were dried at room temperature, and then employed for the experimentations. In order to perform germination tests, 45 of them were placed in glass Petri dishes (20 cm of internal diameters) containing two filter papers saturated with 15 mL of distilled water. Finally, the Petri dishes were incubated for 24 h at 35 °C, in the dark. After the incubation time, the seeds were collected, grinded with mortar and pestle using liquid nitrogen, and then stored at −80 °C for further analyses. The experiment was repeated four more times, in order to have five different biological replicates. Moreover, a parallel experiment, where distilled water was used instead of the biostimulant, was conducted and used as control.

### 4.3. Evaluation of Biometric Parameters under Controlled Conditions

Morphological analysis on seeds germinated in controlled conditions was conducted in order to evaluate differences between biostimulant-treated and untreated seeds. Each seed was weighed and area, perimeter, length, width, and weight were measured by using the SmartGrain software [78], and then statistically evaluated comparing treated and control seeds by a Student’s *t*-test at *p* < 0.05 using SYSTAT 10 software. All measurements were taken immediately after treatment (T_0_) and after 24 h of incubation at 35 °C, in the dark (T_1_). The changes were calculated on values obtained from the difference between T_1_ and T_0_. In addition, germination percentages were calculated for control and biostimulant-treated seeds at 24, 48, and 72 h of incubation at 35 °C, in the dark.

### 4.4. Rna Isolation and Rna-Seq Analysis of Seeds Incubated under Controlled Conditions

#### 4.4.1. Total Rna Isolation

Total RNA was isolated from powdered seeds, as previously described, by using TRIzol^®^ reagent (Thermo Fisher Scientific, Waltham, MA, USA) [79]. Moreover, in order to increase RNA yield, the manufacturer’s protocol was modified according to the suggestion reported by Wang [80]. After isolation, total RNA was purified by using the RNeasy^®^ Mini Kit (Qiagen, Hilden, Germany), following the RNA clean-up protocol. RNA concentration was evaluated by spectrophotometry (Ultrospec 3000, Amersham Pharmacia Biotech, Little Chalfont, UK). Sample quality was checked by using the RNA 6000 Nano kit and the Agilent 2100 Bioanalyzer (Agilent Technologies, San Diego, CA, USA), according to manufacturer’s instructions.

#### 4.4.2. RNA-Seq Analysis

Sequencing libraries were generated using a NEBNext^®^ Ultra^™^ RNA Library Prep Kit for Illumina^®^ (NEB, Ipswich, MA, USA) at Novogene (Hong Kong) and index codes were added to attribute sequences to each sample. The library were sequenced on an Illumina HiseqX platform and 150 bp paired-end reads were generated. Three biological replicates were used for RNA-Seq analysis. Raw data (raw reads) of fastq format were processed and clean reads were obtained by removing reads containing adapter and low-quality reads from raw data, running Sickle (https://github.com/najoshi/sickle), and trimmed for the presence of residual adapter sequences through Scythe (https://github.com/vsbuffalo/scythe). All the downstream analyses were based on the clean data with high quality. Reference genome and gene model annotation files were directly downloaded from Genbank (https://www.ncbi.nlm.nih.gov/genome/?term=glycine+max). The index of the reference genome was built using Bowtie v2.2.3 and paired-end clean reads were aligned to the reference genome using TopHat v2.0.12. We selected TopHat as the mapping tool because it could generate a database of splice junctions based on the gene model annotation file, and thus a better mapping result than other non-splice mapping tools. HTSeq v0.6.1 was used to count the reads numbers mapped to each gene. A similarity matrix of the control and treatment (+biostimulant) data was built up calculating 1 cosine distance (http://amp.pharm.mssm.edu/clustergrammer). Differential expression analysis of two conditions (three biological replicates per condition) was performed using the DESeq R package (1.18.0). DESeq provides statistical routines for determining differential expression in digital gene expression data using a model based on the negative binomial distribution. The resulting *p*-values were adjusted using the Benjamini and Hochberg’s approach for controlling the false discovery rate. Genes with an adjusted *p* < 0.05 found by DESeq were assigned as differentially expressed. A gene ontology (GO) enrichment analysis of differentially expressed genes was implemented using the enrichment term engine (GO terms, KEGG pathways/INTERPRO domain) implemented in STRING (https://string-db.org) and GO terms were visualized with a hierarchical clustering tree function summarizing the correlation among significant GO terms, using ShinyGO (http://bioinformatics.sdstate.edu/go). STRING database collects, scores, and integrate all publicly available sources of protein and protein–protein interaction information. Moreover, it implements many basic services such as GO, KEGG, and INTERPRO enrichment functions together with more complex analyses (interactome). To characterize the function of genes differentially expressed in treated plants, the soybean genes were organized in lists of upregulated and downregulated genes and compared to Arabidopsis proteome (TAIR version 10), using reciprocal blast best hits, to find species orthologs. The list of orthologs was submitted to STRING and enrichments were recorded when terms were more enriched in the set of query proteins than the background, considering a FDR value less than 0.05 [81]. An interactome map was built using DEG (up-/downregulated) based on the STRING^®^ database, with known and predicted protein–protein interactions (PPI) and the networks were built accordingly. The number of observed edges was compared with the expected number of edges, which gives how many edges is to be expected if the nodes are to be selected at random, and PPI enrichment *p*-values were calculated. A small PPI value indicates that the nodes are not random and that the observed number of edges is significant.

### 4.5. Evaluation of Antioxidant Enzyme Activity of Seeds Incubated under Controlled Conditions

Total proteins were extracted, according to a previously reported protocol [41]. Briefly, 500 mg of powdered seeds were used for the extraction, and all the steps were carried out at 4 °C. The extraction buffer included 50 mM sodium phosphate (pH 7.5), 250 mM sucrose, 1.0 mM EDTA, 10 mM KCl, 1 mM MgCl_2_, 0.5 mM phenylmethylsulfonyl fluoride (PMSF), 0.1 mM dithiothreitol (DTT), and 1% (*w*/*v*) polyvinylpolypyrrolidone (PVPP) in a 1:10 (*w*/*v*) proportion. The homogenate was mixed by pipetting, and then centrifuged 20 min at 25,000 g and at 4 °C. The supernatant, containing total proteins, was used for enzymatic assays. Soluble protein content was evaluated by the method of Bradford [82] using bovine serum albumin (BSA) as a standard.

#### 4.5.1. Superoxide Dismutase Activity (Sod, EC 1.15.1.1).

SOD activity was evaluated, as previously described [83]. Briefly, 1 mL containing 50 mM sodium phosphate buffer (pH 7.8), 13 mM methionine, 75 µM nitro blue tetrazolium (NBT), 2 µM riboflavin, and 0.1 mM EDTA was incubated with an appropriate dilution of enzyme extract. The samples were placed 30 cm under a light source (4000 lux) for 15 min. In parallel, two different blanks were prepared, i.e., one without the enzyme extract that was placed under the light and that totally developed the reaction; the second one, containing the enzyme extract but placed in the dark, with the aim to avoid the reaction, and therefore was used as control. The absorbance of both samples and blanks was detected at 560 nm.

#### 4.5.2. Catalase Activity (CAT, EC 1.11.1.6).

Cat Activity was evaluated, as previously described [84]. Briefly, the absorbance at 240 nm was monitored for 120 sec with the aim to evaluate the change due to the decreased absorption of H_2_O_2_ (ɛ = 39.4 mM^−1^ cm^−1^). The 1 mL reaction mixture contained 50 mM sodium phosphate, pH 7.0, 15 mM H_2_O_2_, and enzyme extract. The reaction was started by addition of H_2_O_2_.

#### 4.5.3. Glutathione-S-transferases (GST, EC 2.5.1.18)

The enzyme activity was evaluated by monitoring the absorbance variation at 340 nm for 15 min of the 1-chloro-2,4-dinitrobenzene (CDNB) used as substrate [65]. The assay was performed in 1 mL of reaction solution containing 100 mM potassium phosphate buffer (pH 7.0), 1 mM reduced glutathione (GSH), 1 mM 1-chloro-2,4-dinitro-benzene (CDNB) (10 mM CDNB dissolved in 50% acetone stock solution), and enzyme extract. The reaction was started by adding CDNB.

### 4.6. Evaluation of Non-Protein Thiol Content of Seeds Incubated under Controlled Conditions

The assay was carried out by mixing 500 µL of crude extract, obtained as described before, to 100 µL of 25% (*w*/*v*) trichloroacetic acid (TCA). The samples were centrifuged at 12,000× *g* for 20 min, at 4 °C. Then, 300 µL of supernatant were added to 2.7 mL of 0.6 mM 5,5’- dithiobis (2-nitrobenzoic acid) (DTNB) prepared in 0.1 M sodium phosphate buffer (pH 8.0). The spectrophotometer was used to quantify the sulfhydryl content. The absorbance was detected at 412 nm [65].

### 4.7. Evaluation of Hydrogen Peroxide Levels of Seeds Incubated under Controlled Conditions

The hydrogen peroxide level was detected, according to the protocol described for the first time by Velikova and colleagues [85]. Briefly, powdered seeds (0.5 g) were homogenized with 5 mL of 0.1% (*w*/*v*) TCA. The samples were centrifuged at 12,000 g for 15 min and 0.5 mL of supernatant was added to 0.5 mL 10 mM potassium phosphate buffer (pH 7.0) and 1 mL 1 M KI. The absorbance was read at 390 nm and the H_2_O_2_ content was determined based on a standard curve.

### 4.8. Statistical Analysis

For biometric and antioxidant system analyses, five biological replicates were used for the statistical treatment of the data. Results are expressed as mean values ± standard deviation (SD). Significance of differences observed in datasets was tested by one-way ANOVA followed by Tukey’s test, or by Student’s *t*-test at *p* < 0.05 using SYSTAT 10 software.

## 5. Conclusions

The results of this study displayed how seed pretreatment with the biostimulant had a positive effect on soybean grown under heat stress. In particular, under controlled conditions, this biostimulant was able to enhance the germination rate of soybean seeds incubated at 35 °C, although the effects were visible only at later stages (72 h after imbibition). Furthermore, RNA-Seq analysis carried out on seeds incubated 24 h at 35 °C showed the modulation of about 900 genes (51 upregulated and 828 downregulated). Interestingly, more than one third of the upregulated genes encoded for different methyltransferases, proteins involved in abiotic stress response and in various processes, including DNA repair, a mechanism crucial for a successful seed germination. All of them seemed to act on rRNA raising the hypothesis of a translation-dependent layer of regulation, which deserves to be deeply investigated. Some metabolic pathways, such as carbohydrate metabolism, response to stress, and hormone signaling were negatively regulated by the treatment. This unexpected downregulation might be related to the seed pretreatment activity of the biostimulant to decrease the water uptake in favor of cellular damage prevention. In addition, the antioxidant system activity (ROS scavenging enzymes and non-protein thiol content) evaluated on the same seeds employed for RNA sequencing, revealed a lower amount of hydrogen peroxide in biostimulant-treated seeds, correlated to lower activities and lower expression levels of the corresponding detoxification enzymes, thus confirming the protective action of this biostimulant.

## Figures and Tables

**Table 1:** Biometric parameters of untreated and biostimulant-treated seeds in controlled conditions. Measurements were done before sowing and treatment (T_0_) and at 24 h after incubation at 35 °C, in the dark (T_1_). Values are expressed as mean ± standard deviation (SD) of five different biological replicates, each of which are composed of 45 seeds. For each row, different letters indicate statistically significant differences between untreated and biostimulant-treated samples, as calculated by *t*-test (*p* < 0.05). The last column (%Δ) reports the percentage relative change between untreated and biostimulant-treated samples.

Seed Parameters	Untreated	Biostimulant-Treated	%Δ
Area	84.46 ± 4.91 ^a^	134.80 ± 8.90 ^b^	59.55% ± 1.26%
Perimeter	40.71 ± 2.72 ^a^	58.99 ± 2.71 ^b^	45.04% ± 3.04%
Length	55.62 ± 2.74 ^a^	76.01 ± 3.35 ^b^	36.68% ± 0.71%
Width	13.08 ± 1.55 ^a^	27.82 ± 3.17 ^b^	112.77% ± 0.98%
Weight	101.86 ± 2.33 ^a^	102.50 ± 3.49 ^a^	0.61% ± 0.02%

**Table 2:** Germination percentage of untreated and biostimulant-treated soybean seeds incubated at 35 °C in the dark. Values are expressed as mean ± standard deviation (SD) of five different biological replicates, each of which are composed of 45 seeds. For each row, different letters indicate significant differences between untreated and biostimulant-treated samples, as calculated by *t*-test (*p* < 0.05). The last column (%Δ) reports the percentage relative change between untreated and biostimulant-treated samples.

	Untreated	Biostimulant-Treated	%Δ
24 h	n.d.	n.d.	-
48 h	68.80 ± 1.16 ^a^	77.70 ± 0.58 ^b^	12.95% ± 1.06%
72 h	82.22 ± 0.58 ^a^	91.13 ± 0.55 ^b^	10.84% ± 0.11%

**Table 3:** List of the selected GO of the genes upregulated after the treatment with the biostimulant. The table reports the pathway ID or GO category, the count in gene set, and the false discovery rate (FDR) for each gene superfamily (FDR < 0.05).

Pathway ID GO Category	Pathway Description	Count in Gene Set	FDR
**Upregulated Genes**			
IPR029063	SAM-dependent methyltransferase	9	6.7 × e^−08^
IPR040072	Methyltransferases (Class A)	3	8.8 × e^−05^
IPR004383	Ribosomal RNA large subunit methyltransferase RlmN/Cfr	3	5.73 × e^−06^
IPR027492	Dual-specificity RNA methyltransferase RlmN	3	0.332
IPR015507	Ribosomal RNA large subunit methyltransferase E	2	0.00509
IPR002877	Ribosomal RNA methyltransferase FtsJ domain	2	0.00847
IPR023267	RNA (C5-cytosine) methyltransferase	2	0.0263
IPF01728	FtsJ-like methyltransferase	2	0.0149
IPR006638	Elongator protein 3/MiaB/NifB	2	0.0106
IPR001678	SAM-dependent methyltransferase RsmB/NOP2-type	2	0.0304
**Downregulated Genes**			
GO:0042221	Response to chemical	90	8.83 × e^−11^
GO:0050896	Response to stimulus	135	2.24 × e^−10^
GO:0044699	Single-organism process	186	9.73 × e^−09^
GO:0008152	Metabolic process	211	1.21 × e^−07^
GO:0044763	Single-organism cellular process	154	2.49 × e^−07^
GO:0009987	Cellular process	213	3.13 × e^−06^
GO:0006950	Response to stress	80	5.49 × e^−06^
GO:0010033	Response to organic substance	60	1.13 × e^−05^
GO:0071704	Organic substance metabolic process	175	2.30 × e^−05^
GO:0009628	Response to abiotic stimulus	52	3.51 × e^−05^
GO:0044238	Primary metabolic process	160	0.000933
GO:0005975	Carbohydrate metabolic process	35	0.00117
GO:0009755	Hormone-mediated signaling pathway	34	0.00117
GO:0009058	Biosynthetic process	100	0.00211
GO:0006952	Defense response	39	0.00228
GO:1901698	Response to nitrogen compound	14	0.00244
GO:0007165	Signal transduction	42	0.00993
GO:0071554	Cell wall organization or biogenesis	21	0.00993

SAM = S-adenosylmethionine.

**Table 4:** List of the upregulated genes involved in ribosomal RNA (rRNA) methylation and ribosome assembly.

Soybean Locus	Arabidopsis Locus	Category	Annotation
LOC100780381	AT1G01860	rRNA methylation	mRNA (2’-O-methyladenosine-N6-)-methyltransferase activity, rRNA (adenine-N6,N6-)-dimethyltransferase activity
LOC100796633	AT1G54310	rRNA methylation	RNA binding
LOC100813692	AT1G60230	rRNA methylation	rRNA base methylation, tRNA methylation
LOC100818638	AT2G39670	rRNA methylation	rRNA base methylation, tRNA methylation
LOC100779327	AT3G13180	rRNA methylation	rRNA (cytosine-C5-)-methyltransferase activity
LOC100804869	AT3G19630	rRNA methylation	rRNA base methylation, tRNA methylation
LOC100778294	AT3G57000	rRNA methylation	rRNA (pseudouridine) methyltransferase activity, rRNA binding
LOC100807160	AT4G26600	rRNA methylation	S-adenosylmethionine-dependent methyltransferase activity, rRNA (cytosine-C5-)-methyltransferase activity
LOC100809923	AT5G01230	rRNA/tRNA methylation	RNA methyltransferase activity, tRNA methyltransferase activity
LOC100787277	AT5G10910	rRNA methylation	Plastid rRNA methyltransferase involved in ribosome biogenesis and plant development. Accounts to the N4-methylation of C1352 in chloroplast 16S rRNA.
LOC100805818	AT5G13830	rRNA methylation	RNA methyltransferase activity, rRNA (uridine-2’-O-)-methyltransferase activity
LOC100818428	AT5G50110	rRNA methylation	rRNA (guanine-N7-)-methyltransferase activity, rRNA methyltransferase activity
LOC100804672	AT1G12650	Ribosome assembly	Cleavage involved in rRNA processing, maturation of SSU-rRNA from tricistronic rRNA transcript (SSU-rRNA, 5.8S rRNA, LSU-rRNA)
LOC100798373	AT5G20600	Ribosome assembly	rRNA processing
LOC100802650	AT5G38720	Ribosome assembly	rRNA processing, ribosomal small subunit assembly
LOC100808860	AT5G40530	Ribosome assembly	Methylated histone binding involved in rDNA heterochromatin assembly
LOC100816856	AT5G08420	Ribosome assembly	Small subunit processome (nucleolus, nucleus)

## Supplementary Materials

The following materials are available online at https://www.mdpi.com/2223-7747/9/10/1308/s1, Supplementary Table S1: Plant height (cm), root length (cm), pod number per plant (number), total seed weight (g), and final yield (Kg ha^−1^) of soybean sown in field trials in 2017 at Detec Experimental Station, São Paolo State, Taquarituba, Supplementary Table S2: Tukey’s HSD post hoc differences in plant height, root length, pod number/plant, TSW, and final yield, Supplementary Table S3: List of the upregulated and downregulated genes analyzed by RNA-Seq, Supplementary Table S4: Individual biochemical measurement of H_2_O_2_ (nmol g^−1^ FW), SOD (nKat mg^−1 ^prot), CAT (μKat mg^−1^ prot), GST (μKat mg^−1^ prot), non-protein thiols (μmol g−1 FW) evaluated on soybean untreated or treated with the biostimulant, Supplementary Table S5: Tukey’s HSD post hoc differences in the relative measures of H_2_O_2_ (nmol g^−1^ FW), SOD (nKat mg^−1^ prot), CAT (μKat mg^−1^ prot), GST (μKat mg^−1^ prot), non-protein thiols (μmol g^−1^ FW), Supplementary Figure S1: Volcano plot, PCA, and heatmap of RNA-Seq analyses.

## References

[1] Pagano M.C., Miransari M. (2016). Production Worldwide.

[2] Wingeyer A.B., Amado T.J.C., Pérez-Bidegain M., Studdert G.A., Perdomo Varela C.H., Garcia F.O., Karlen D.L. (2015). Soil quality impacts of current South American agricultural practices. Sustainability.

[3] Hasanuzzaman M., Nahar K., Alam M.M., Roychowdhury R., Fujita M. (2013). Physiological, biochemical, and molecular mechanisms of heat stress tolerance in plants. Int. J. Mol. Sci..

[4] Chebrolu K.K., Fritschi F.B., Ye S., Krishnan H.B., Smith J.R., Gillman J.D. (2016). Impact of heat stress during seed development on soybean seed metabolome. Metabolomics.

[5] Teixeira E.I., Fischer G., Van Velthuizen H., Walter C., Ewert F. (2013). Global hot-spots of heat stress on agricultural crops due to climate change. Agric. For. Meteorol..

[6] Probert R.J. (2000). The role of temperature in the regulation of seed dormancy and germination. Seeds Ecol. Regen. Plant Communities.

[7] Jisha K.C., Vijayakumari K., Puthur J.T. (2013). Seed priming for abiotic stress tolerance: An overview. Acta Physiol. Plant..

[8] Ashraf M., Foolad M.R. (2005). Pre-Sowing Seed Treatment-A Shotgun Approach to Improve Germination, Plant Growth, and Crop Yield Under Saline and Non-Saline Conditions. Adv. Agron..

[9] Campobenedetto C., Grange E., Mannino G., Van Arkel J., Beekwilder J., Karlova R., Garabello C., Contartese V., Bertea C.M. (2020). A Biostimulant Seed Treatment Improved Heat Stress Tolerance During Cucumber Seed Germination by Acting on the Antioxidant System and Glyoxylate Cycle. Front. Plant Sci..

[10] Ziosi V., Zandoli R., Vitali F., Di Nardo A. (2012). Folicist^®^, a biostimulant based on acetyl-thioproline, folic acid and plant extracts, improves seed germination and radicle extension. Acta Hortic..

[11] Pichyangkura R., Chadchawan S. (2015). Biostimulant activity of chitosan in horticulture. Sci. Hortic..

[12] Masondo N.A., Kulkarni M.G., Finnie J.F., Van Staden J. (2018). Influence of biostimulants-seed-priming on Ceratotheca triloba germination and seedling growth under low temperatures, low osmotic potential and salinity stress. Ecotoxicol. Environ. Saf..

[13] Kavipriya R., Dhanalakshmi P.K., Jayashree S., Thangaraju N. (2011). Seaweed extract as a biostimulant for legume crop, green gram. J. Ecobiotechnol..

[14] Qiu Y., Amirkhani M., Mayton H., Chen Z., Taylor A.G. (2020). Biostimulant seed coating treatments to improve cover crop germination and seedling growth. Agronomy.

[15] Bulgari R., Franzoni G., Ferrante A. (2019). Biostimulants Application in Horticultural Crops under Abiotic Stress Conditions. Agronomy.

[16] Du Jardin P. (2015). Plant biostimulants: Definition, concept, main categories and regulation. Sci. Hortic..

[17] Mannino G., Nerva L., Gritli T., Novero M., Fiorilli V., Bacem M., Bertea C.M., Lumini E., Chitarra W., Balestrini R. (2020). Effects of Different Microbial Inocula on Tomato Tolerance to Water Deficit. Agronomy.

[18] Petropoulos S.A., Fernandes Â., Plexida S., Chrysargyris A., Tzortzakis N., Barreira J.C.M., Barros L., Ferreira I.C.F.R. (2020). Biostimulants application alleviates water stress effects on yield and chemical composition of greenhouse green bean (Phaseolus vulgaris L.). Agronomy.

[19] Rouphael Y., Colla G. (2018). Synergistic biostimulatory action: Designing the next generation of plant biostimulants for sustainable agriculture. Front. Plant Sci..

[20] Rouphael Y., Lucini L., Miras-Moreno B., Colla G., Bonini P., Cardarelli M. (2020). Metabolomic Responses of Maize Shoots and Roots Elicited by Combinatorial Seed Treatments With Microbial and Non-microbial Biostimulants. Front. Microbiol..

[21] De Pádua G.P., França-Neto J.D.B., De Carvalho M.L.M., Krzyzanowski F.C., Guimarães R.M. (2009). Incidence of green soybean seeds as a function of environmental stresses during seed maturation. Rev. Bras. Sementes.

[22] Hulme M., Sheard N. (1999). Climate Change Scenarios for Brazil.

[23] Ferreira D.B., Rao V.B. (2011). Recent climate variability and its impacts on soybean yields in Southern Brazil. Theor. Appl. Climatol..

[24] Struck A.W., Thompson M.L., Wong L.S., Micklefield J. (2012). S-Adenosyl-Methionine-Dependent Methyltransferases: Highly Versatile Enzymes in Biocatalysis, Biosynthesis and Other Biotechnological Applications. ChemBioChem.

[25] Wang S.C., Frey P.A. (2007). S-adenosylmethionine as an oxidant: The radical SAM superfamily. Trends Biochem. Sci..

[26] Weitbrecht K., Müller K., Leubner-Metzger G. (2011). First off the mark: Early seed germination. J. Exp. Bot..

[27] Luo X., Wang Y., Li Q., Wang D., Xing C., Zhang L., Xu T., Fang F., Wang F. (2018). Accumulating mechanism of γ-aminobutyric acid in soybean (Glycine max L.) during germination. Int. J. Food Sci. Technol..

[28] Taylor A.G., Allen P.S., Bennett M.A., Bradford K.J., Burris J.S., Misra M.K. (1998). Seed enhancements. Seed Sci. Res..

[29] Reddy P.P. (2015). Climate Resilient Agriculture for Ensuring Food Security.

[30] Savvides A., Ali S., Tester M., Fotopoulos V. (2016). Chemical priming of plants against multiple abiotic stresses: Mission possible?. Trends Plant Sci..

[31] Lima J.J.P., Buitink J., Lalanne D., Rossi R.F., Pelletier S., Da Silva E.A.A., Leprince O. (2017). Molecular characterization of the acquisition of longevity during seed maturation in soybean. PLoS ONE.

[32] Ali Q., Perveen R., El-Esawi M.A., Ali S., Hussain S.M., Amber M., Iqbal N., Rizwan M., Alyemeni M.N., El-Serehy H.A. (2020). Low Doses of Cuscuta reflexa Extract Act as Natural Biostimulants to Improve the Germination Vigor, Growth, and Grain Yield of Wheat Grown under Water Stress: Photosynthetic Pigments, Antioxidative Defense Mechanisms, and Nutrient Acquisition. Biomolecules.

[33] Ibrahim E.A. (2016). Seed priming to alleviate salinity stress in germinating seeds. J. Plant Physiol..

[34] Wang X., Cai J., Liu F., Dai T., Cao W., Wollenweber B., Jiang D. (2014). Multiple heat priming enhances thermo-tolerance to a later high temperature stress via improving subcellular antioxidant activities inwheat seedlings. Plant Physiol. Biochem..

[35] Savy D., Cozzolino V., Nebbioso A., Drosos M., Nuzzo A., Mazzei P., Piccolo A. (2016). Humic-like bioactivity on emergence and early growth of maize (Zea mays L.) of water-soluble lignins isolated from biomass for energy. Plant Soil.

[36] Amirkhani M., Netravali A.N., Huang W., Taylor A.G. (2016). Investigation of soy protein–based biostimulant seed coating for broccoli seedling and plant growth enhancement. HortScience.

[37] Singh R.K., Deshmukh R., Muthamilarasan M., Rani R., Prasad M. (2020). Versatile roles of aquaporin in physiological processes and stress tolerance in plants. Plant Physiol. Biochem..

[38] Chen K., Fessehaie A., Arora R. (2013). Aquaporin expression during seed osmopriming and post-priming germination in spinach. Biol. Plant.

[39] Noodén L.D., Blakley K.A., Grzybowski J.M. (1985). Control of Seed Coat Thickness and Permeability in Soybean. Plant Physiol..

[40] Nair P., Kandasamy S., Zhang J., Ji X., Kirby C., Benkel B., Hodges M.D., Critchley A.T., Hiltz D., Prithiviraj B. (2012). Transcriptional and metabolomic analysis of Ascophyllum nodosum mediated freezing tolerance in Arabidopsis thaliana. BMC Genom..

[41] Contartese V., Garabello C., Occhipinti A., Barbero F., Bertea C.M. Effects of a new biostimulant on gene expression and metabolic responses of tomato plants. Proceedings of the II World Congress on the Use of Biostimulants in Agriculture 1148.

[42] Tirnaz S., Batley J. (2019). DNA Methylation: Toward Crop Disease Resistance Improvement. Trends Plant Sci..

[43] Conrath U. (2011). Molecular aspects of defence priming. Trends Plant Sci..

[44] Kim J.M., Sasaki T., Ueda M., Sako K., Seki M. (2015). Chromatin changes in response to drought, salinity, heat, and cold stresses in plants. Front. Plant Sci..

[45] Hu J., Manduzio S., Kang H. (2019). Epitranscriptomic RNA methylation in plant development and abiotic stress responses. Front. Plant Sci..

[46] Genuth N.R., Barna M. (2018). The discovery of ribosome heterogeneity and its implications for gene regulation and organismal life. Mol. Cell.

[47] Basu A., Das P., Chaudhuri S., Bevilacqua E., Andrews J., Barik S., Hatzoglou M., Komar A.A., Mazumder B. (2011). Requirement of rRNA methylation for 80S ribosome assembly on a cohort of cellular internal ribosome entry sites. Mol. Cell. Biol..

[48] Schosserer M., Minois N., Angerer T.B., Amring M., Dellago H., Harreither E., Calle-Perez A., Pircher A., Gerstl M.P., Pfeifenberger S. (2015). Methylation of ribosomal RNA by NSUN5 is a conserved mechanism modulating organismal lifespan. Nat. Commun..

[49] Liberman N., O’Brown Z.K., Earl A.S., Boulias K., Gerashchenko M.V., Wang S.Y., Fritsche C., Fady P.-E., Dong A., Gladyshev V.N. (2020). N6-adenosine methylation of ribosomal RNA affects lipid oxidation and stress resistance. Sci. Adv..

[50] Lisso J., Altmann T., Müssig C. (2006). The AtNFXL1 gene encodes a NF-X1 type zinc finger protein required for growth under salt stress. FEBS Lett..

[51] Sharma M., Pandey G.K. (2016). Expansion and function of repeat domain proteins during stress and development in plants. Front. Plant Sci..

[52] Laluk K., Abuqamar S., Mengiste T. (2011). The arabidopsis mitochondria-localized pentatricopeptide repeat protein PGN functions in defense against necrotrophic fungi and abiotic stress tolerance. Plant Physiol..

[53] Li P.S., Yu T.F., He G.H., Chen M., Zhou Y.-B., Chai S.C., Xu Z.S., Ma Y.Z. (2014). Genome-wide analysis of the Hsf family in soybean and functional identification of GmHsf-34 involvement in drought and heat stresses. BMC Genom..

[54] Ikeda M., Mitsuda N., Ohme-Takagi M. (2011). Arabidopsis HsfB1 and HsfB2b act as repressors of the expression of heat-inducible Hsfs but positively regulate the acquired thermotolerance. Plant Physiol..

[55] Demidchik V. (2015). Mechanisms of oxidative stress in plants: From classical chemistry to cell biology. Environ. Exp. Bot..

[56] Torres M.A., Dangl J.L., Jones J.D.G. (2002). Arabidopsis gp91phox homologues Atrbohd and Atrbohf are required for accumulation of reactive oxygen intermediates in the plant defense response. Proc. Natl. Acad. Sci. USA.

[57] Bailly C. (2004). Active oxygen species and antioxidants in seed biology. Seed Sci. Res..

[58] Wojtyla Ł., Lechowska K., Kubala S., Garnczarska M. (2016). Different modes of hydrogen peroxide action during seed germination. Front. Plant Sci..

[59] Yi H., Galant A., Ravilious G.E., Preuss M.L., Jez J.M. (2010). Sensing sulfur conditions: Simple to complex protein regulatory mechanisms in plant thiol metabolism. Mol. Plant.

[60] Szalai G., Kellős T., Galiba G., Kocsy G. (2009). Glutathione as an antioxidant and regulatory molecule in plants under abiotic stress conditions. J. Plant Growth Regul..

[61] Kocsy G., Szalai G., Galiba G. (2002). Induction of glutathione synthesis and glutathione reductase activity by abiotic stresses in maize and wheat. Sci. World J..

[62] Peltzer D., Dreyer E., Polle A. (2002). Differential temperature dependencies of antioxidative enzymes in two contrasting species: Fagus sylvatica and Coleus blumei. Plant Physiol. Biochem..

[63] Nieto-Sotelo J., Ho T.-H.D. (1986). Effect of heat shock on the metabolism of glutathione in maize roots. Plant Physiol..

[64] Nahar K., Hasanuzzaman M., Alam M.M., Fujita M. (2015). Exogenous glutathione confers high temperature stress tolerance in mung bean (Vigna radiata L.) by modulating antioxidant defense and methylglyoxal detoxification system. Environ. Exp. Bot..

[65] Jain M., Bhalla-Sarin N. (2001). Glyphosate-induced increase in glutathione S-transferase activity and glutathione content in groundnut (Arachis hypogaea L.). Pestic. Biochem. Physiol..

[66] Zagorchev L., Seal C.E., Kranner I., Odjakova M. (2013). A central role for thiols in plant tolerance to abiotic stress. Int. J. Mol. Sci..

[67] Turk H., Erdal S., Genisel M., Atici O., Demir Y., Yanmis D. (2014). The regulatory effect of melatonin on physiological, biochemical and molecular parameters in cold-stressed wheat seedlings. Plant Growth Regul..

[68] Ali M., Hayat S., Ahmad H., Ghani M.I., Amin B., Atif M.J., Cheng Z. (2019). Priming of Solanum melongena L. seeds enhances germination, alters antioxidant enzymes, modulates ROS, and improves early seedling growth: Indicating aqueous garlic extract as seed-priming bio-stimulant for eggplant production. Appl. Sci..

[69] Bellieny-Rabelo D., De Oliveira E.A.G., Da Silva Ribeiro E., Pessoa Costa E., Oliveira A.E.A., Venancio T.M. (2016). Transcriptome analysis uncovers key regulatory and metabolic aspects of soybean embryonic axes during germination. Sci. Rep..

[70] Winter H., Huber S.C. (2000). Regulation of sucrose metabolism in higher plants: Localization and regulation of activity of key enzymes. Crit. Rev. Biochem. Mol. Biol..

[71] Fernandez O., Béthencourt L., Quero A., Sangwan R.S., Clément Christophe C. (2010). Trehalose and plant stress responses: Friend or foe?. Trends Plant Sci..

[72] De La Torre F., Cañas R.A., Pascual M.B., Avila C., Cánovas F.M. (2014). Plastidic aspartate aminotransferases and the biosynthesis of essential amino acids in plants. J. Exp. Bot..

[73] Balestrazzi A., Confalonieri M., Macovei A., Donà M., Carbonera D. (2011). Genotoxic stress and DNA repair in plants: Emerging functions and tools for improving crop productivity. Plant Cell Rep..

[74] Han C., Yang P. (2015). Studies on the molecular mechanisms of seed germination. Proteomics.

[75] Shuai H., Meng Y., Luo X., Chen F., Zhou W., Dai Y., Qi Y., Du J., Yang F., Liu J. (2017). Exogenous auxin represses soybean seed germination through decreasing the gibberellin/abscisic acid (GA/ABA) ratio. Sci. Rep..

[76] Henning A.A. (2005). Patologia e Tratamento de Sementes: Noções Gerais.

[77] Dos Santos S.F., Carvalho E.R., Rocha D.K., Nascimento R.M. (2018). Composition and volumes of slurry in soybean seeds treatment in the industry and physiological quality during storage. J. Seed Sci..

[78] Tanabata T., Shibaya T., Hori K., Ebana K., Yano M. (2012). SmartGrain: High-throughput phenotyping software for measuring seed shape through image analysis. Plant Physiol..

[79] Mannino G., Perrone A., Campobenedetto C., Schittone A., Margherita Bertea C., Gentile C. (2020). Phytochemical profile and antioxidative properties of Plinia trunciflora fruits: A new source of nutraceuticals. Food Chem..

[80] Wang G., Wang G., Zhang X., Wang F., Song R. (2012). Isolation of high quality RNA from cereal seeds containing high levels of starch. Phytochem. Anal..

[81] Szklarczyk D., Gable A.L., Lyon D., Junge A., Wyder S., Huerta-Cepas J., Simonovic M., Doncheva N.T., Morris J.H., Bork P. (2019). STRING v11: Protein–protein association networks with increased coverage, supporting functional discovery in genome-wide experimental datasets. Nucleic Acids Res..

[82] Bradford M.M. (1976). A rapid and sensitive method for the quantitation of microgram quantities of protein utilizing the principle of protein-dye binding. Anal. Biochem..

[83] Krishnan N., Chattopadhyay S., Kundu J.K., Chaudhuri A. (2002). Superoxide dismutase activity in haemocytes and haemolymph of Bombyx mori following bacterial infection. Curr. Sci..

[84] Goth L. (1991). A simple method for determination of serum catalase activity and revision of reference range. Clin. Chim. Acta.

[85] Velikova V., Yordanov I., Edreva A. (2000). Oxidative stress and some antioxidant systems in acid rain-treated bean plants: Protective role of exogenous polyamines. Plant Sci..

