# Supplementary material for: Transcriptome Analyses and Antioxidant Activity Profiling Reveal the Role of a Lignin-Derived Biostimulant Seed Treatment in Enhancing Heat Stress Tolerance in Soybean
Source: Plants (Basel). 2020 Oct 2;9(10):1308. doi: 10.3390/plants9101308 (PMC7601093; doi:10.3390/plants9101308)
Supplement: Supplementary file 1 [file plants-09-01308-s001.pdf]

Supplementary Figure 1: Volcano plot (A) displaying differential expressed genes between biostimulant-treated and untreated seeds. The y-axis corresponds to the mean expression value of $\log_{10}$ (q–value), and the x-axis displays the $\log_2$ fold change value. The red dots represent the up-regulated expressed transcripts, meanwhile the green dots represent the transcripts whose expression was down-regulated. Positive x-values represent up-regulation and negative x-values represent down-regulation. (**B**) and (**C**) respectively show the PCA and heatmap to visualize the separate clustering of treated and control samples.

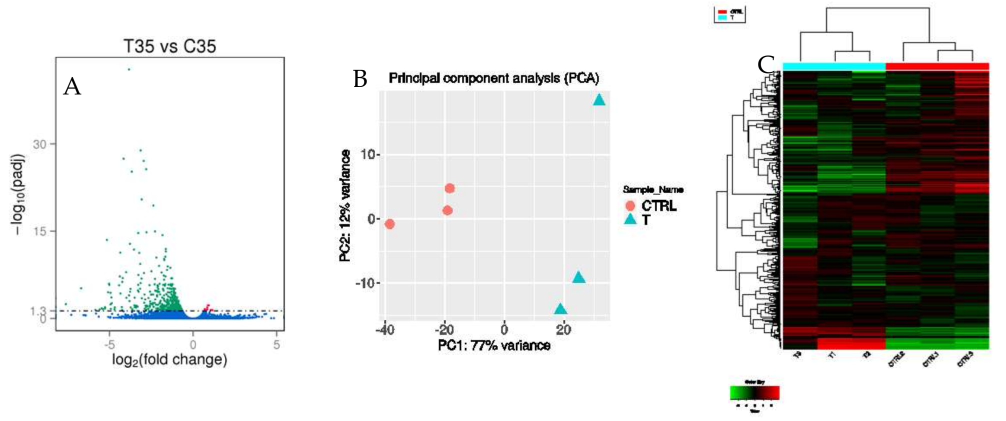

Supporting Table 1: Field experiments were conducted during the hot season (December – March) of 2017 at Detec Experimental Station, São Paolo State, Taquarituba, 23°31'33.7"S 49°15'16.6"W. During the hot season, the temperature at the Detec Experimental Station rises above 35°C. Precipitation averages 1216 mm annually and it is distributed evenly throughout the year. The composition of the soil at the experimental area is 35% sand, 10% silt, and 55% clay. Moreover, its pH equals 4.9, and its carbon and nitrogen percentage is 0.66% and 0.06%, respectively. It contains calcium (Ca) 0.9 comolc kg^{-1}, magnesium (Mg) 0.9 comolc kg^{-1}, aluminum (Al) 0.1 comolc kg^{-1}, phosphorous (P) 2.2 mg kg^{-1}, potassium (K) 125.3 mg kg^{-1}, copper (Cu) 4.3 mg kg^{-1}, zinc (Zn) 1.2 mg kg^{-1}, iron (Fe) 51.2 mg kg^{-1}, manganese (Mn) 46 mg kg^{-1}. Before sowing, soybean seeds were treated with 200 mL of the biostimulant solution diluted 1:3 (*v*/*v*) with distilled water in order to reach the final volume of 800 mL. The diluted biostimulant solution was then added drop by drop to 2.500 g of dried seeds kept in continuous shaking until the complete and visible distribution of the product on the seed surface was obtained. After the treatment, the seeds were dried at room temperature, and then employed for the sowing. Soybean seeds were sown in the experimental field at 1.5 m long rows spaced 0.25 m apart and a depth of 2.5 to 3.5 cm. In order to evaluate the potential effects of the biostimulant, parallel sowing was carried out by treating the seeds simply with water.

Parameters	Untreated	Biostimulant-treated	%Δ
Plant Height (cm)	17.02 ± 0.39^{a}	19.89 ± 0.41^{b}	16.86% ± 0.27%
Root Length (cm)	4.98 ± 0.17^{a}	6.23 ± 0.09^{b}	25.16% ± 2.47%
Pod Number/plant (number)	42.11 ± 0.65^{a}	65.55 ± 1.67^{b}	55.65% ± 1.56%
TSW (g)	120.01 ± 1.46^{a}	139.66 ± 3.12^{b}	16.36% ± 1.18%
Yield (Kg ha^{-1})	3565.12 ± 117.64^{a}	6089.22 ± 157.73^{b}	70.83% ± 1.21%

Plant Height and Root Length were evaluated at 7 days after sowing. Pod Number per Plant, Total Seed Weight and Final Yield were evaluated at harvest time (60 days after sowing). Data are expressed

as means ± standard deviation. For each row, different lowercase letters indicate significant differences as measured by *t*-test ($p < 0.05$) between untreated and biostimulant-treated samples. For additional statistical information, see Supplementary Table 2. The last column (%Δ) reports the percentage relative change between treated sample and untreated.

Supplementary Table 2: Tukey's HSD post hoc differences in the relative measures of Plant Height, Root Length, Pod Number/Plant, TSW and Final Yield. * $p < 0.05$; ** $p < 0.01$; *** $p < 0.005$.

		HSD
Plant Height	Root Length	0.08238
	Pod Number/Plant	0.38801**
	TSW	0.00489
	Final Yield	0.53937**
Root Length	Pod Number/Plant	0.30563***
	TSW	0.08727
	Final Yield	0.45699**
Pod Number/Plant	TSW	0.39292***
	Final Yield	0.15136
TSW	Final Yield	0.54426**

Supplementary Table 4: Individual biochemical measurement of H_2O_2 (nmol g^{-1} FW), SOD (nKat mg^{-1} prot), CAT (μKat mg^{-1} prot), GST (μKat mg^{-1} prot), Non-Protein Thiols (μmol g^{-1} FW) evaluated on soybean untreated or treated with the biostimulant. Absolute measurements were used for the calculation of Relative Measures displayed in Figure 3. For each row, different lowercase letters indicate significant differences as measured by *t*-test ($p < 0.05$) between KIEM®-treated samples and untreated.

Parameters	Untreated	KIEM®-treated
H_2O_2 *(nmol g⁻¹ FW)*	118.11 ± 14.78^a	71.88 ± 10.86^b
SOD *(nKat mg⁻¹ prot)*	315.15 ± 10.89^a	346.02 ± 14.15^a
CAT *(μKat mg⁻¹ prot)*	9.41 ± 0.69^a	2.63 ± 0.20^b
GST *(μKat mg⁻¹ prot)*	88.42 ± 2.32^a	86.11 ± 3.31^a
Non-Protein thiols *(μmol g⁻¹ FW)*	0.42 ± 0.186^a	1.014 ± 0.07^b

Supplementary Table 5: Tukey's HSD post hoc differences in the relative measures of H_2O_2 (nmol g^{-1} FW), SOD (nKat mg^{-1} prot), CAT (μKat mg^{-1} prot), GST (μKat mg^{-1} prot), Non-Protein Thiols (μmol g^{-1} FW). *$p < 0.05$; **$p < 0.01$; ***$p < 0.005$.

		HSD
H_2O_2	SOD	0.48
	CAT	0.32
	GST	0.36
	non-protein thiols	1.76***
SOD	CAT	0.81***
	GST	0.12
	non-protein thiols	1.27***
CAT	GST	0.69**
	non-protein thiols	2.09***
GST	non-protein thiols	1.4***
